# SBILib: a handle for protein modeling and engineering

**DOI:** 10.1093/bioinformatics/btad613

**Published:** 2023-10-05

**Authors:** Patrick Gohl, Jaume Bonet, Oriol Fornes, Joan Planas-Iglesias, Narcís Fernandez-Fuentes, Baldo Oliva

**Affiliations:** Department of Medicine and Life Sciences, SBI-GRIB, Universitat Pompeu Fabra, 08003 Barcelona, Catalonia, Spain; Department of Medicine and Life Sciences, SBI-GRIB, Universitat Pompeu Fabra, 08003 Barcelona, Catalonia, Spain; Department of Medical Genetics, Centre for Molecular Medicine and Therapeutics, BC Children’s Hospital Research Institute, University of British Columbia, Vancouver, BC V5Z 4H4, Canada; Loschmidt Laboratories, Department of Experimental Biology and RECETOX, Faculty of Science, Masaryk University, 625 00 Brno, Czech Republic; International Clinical Research Center, St Anne’s University Hospital Brno, 656 916 Brno, Czech Republic; Institute of Biological, Environmental and Rural Science, Aberystwyth University, Aberystwyth SY23 3DA, United Kingdom; Department of Medicine and Life Sciences, SBI-GRIB, Universitat Pompeu Fabra, 08003 Barcelona, Catalonia, Spain

## Abstract

**Summary:**

The SBILib Python library provides an integrated platform for the analysis of macromolecular structures and interactions. It combines simple 3D file parsing and workup methods with more advanced analytical tools. SBILib includes modules for macromolecular interactions, loops, super-secondary structures, and biological sequences, as well as wrappers for external tools with which to integrate their results and facilitate the comparative analysis of protein structures and their complexes. The library can handle macromolecular complexes formed by proteins and/or nucleic acid molecules (i.e. DNA and RNA). It is uniquely capable of parsing and calculating protein super-secondary structure and loop geometry. We have compiled a list of example scenarios which SBILib may be applied to and provided access to these within the library.

**Availability and implementation:**

SBILib is made available on Github at https://github.com/structuralbioinformatics/SBILib.

## 1 Introduction

Macromolecular structure and interaction profiles are essential for the understanding of protein function in health and disease (Yue *et al.* 2005). Binding affinity may be measured at the interface of protein–protein and protein–DNA interaction (Ma *et al.* 2002), and therefore these sites are diagnostic of interaction stability. On the therapeutic front, protein loop similarity offers the potential for grafting, yielding acceptor proteins with beneficial properties (Jones *et al.* 1986, Tang *et al.* 2019). When the loop presented by one protein is replaced by a biologically active loop of another protein (“loop grafting”) it is possible to transfer that loop's function, if biological activity of the loop is maintained by ensuring similar loop geometry or flexibility (Smith *et al.* 1995, Schenkmayerova *et al.* 2021). These methods, and many more, rely on standalone bioinformatic packages and databases such as ArchDB (Bonet *et al.* 2014a,b), or on methods that require adapting available bioinformatics packages to purpose written code for analysis [e.g. Biopython (Cock *et al.* 2009), pdb-tools (Rodrigues *et al.* 2018), etc.]. In addition to handling 3D structures, these packages must also be able to handle amino acid sequences and secondary structures (i.e. sequence and local regular conformation), in particular for their alignment and comparison. We have developed a Python package to address these functionalities, as well as introduce super-secondary structure (structures composed of one or more adjacent regular secondary structures) handling functionality. The Structural BioInformatics library (SBILib) is designed to facilitate analysis of macromolecular structures and interactions, as well as to integrate the results from external tools such as BLAST (Camacho *et al.* 2009), DSSP (Kabsch and Sander 1983, Touw *et al.* 2015), or CD-HIT (Fu *et al.* 2012) for protein sequence analyses. Its design strength lies in providing several common analysis tools under one umbrella, resulting in a streamlined approach to structural bioinformatics projects, including the prediction of loop conformations (Fernandez-Fuentes *et al.* 2006), redesign of super-secondary protein structures (Bonet *et al.* 2014a,b), quality assessment of protein folds (Aguirre-Plans *et al.* 2021), or analyses of protein–protein and protein–DNA interactions. For example, SBILib is a core component of ArchDB, Frag’r’Us (Bonet *et al.* 2014a,b), MODPIN (Meseguer *et al.* 2020), InteractoMIX (Mirela-Bota *et al.* 2021), and ModCRE (Fornes *et al.* 2022). We would like to place particular emphasis on the extended functionality of SBILib as it compares to other Python packages such as atomium (Ireland and Martin 2020), ProDy (Zhang *et al.* 2021), Biotite (Kunzmann *et al.* 2023) with regards to protein modeling and engineering. Here, we present this Python library which we have made available on our GitHub repository along with a user manual and tutorial.

## 2 SBILib architecture

### 2.1 Installation

The SBILib library can be installed through pip (pip install SBILib) or downloaded from GitHub. Installation instructions are provided in the README file. SBILib dependencies include: BLAST, CD-HIT, DSSP, NumPy (Harris *et al.* 2020), and SciPy (Virtanen *et al.* 2020). After installation, SBILib can be used both interactively through the Python interpreter or the command line in custom scripts.

### 2.2 Input parsing

The library is designed to process protein and nucleic acid (i.e. DNA and RNA) structural information both in Protein Data Bank (PDB) and *mmCIF* formats. This information can be retrieved directly from the PDB (Berman *et al.* 2000) via accession codes or the PDBlink module provided within SBILib, or from a local file. The PDBlink module also enables the automatic retrieval and management of PDB files locally. Additionally UniProt IDs may be used to retrieve models from the AlphaFold Protein Structure Database (Varadi *et al.* 2022) using the AlphaFoldlink module.

### 2.3 Integration of external software

The library can handle the results from external software (BLAST, CD-HIT, DSSP) and integrates them as internal objects or methods. External software must be installed locally, and users can manually specify their system address in the configuration file (SBILib/external/configSBI.txt).

## 3 SBILib capabilities

### 3.1 Parsing

SBILib provides PDB, DSSP, and BLAST parsing. The SBILib’s BLAST module takes amino acid sequences as input to search for potential homologs. The results are stored as a Python object. Automatic parsing and storage of BLAST results are handled by additional modules. In addition to filtering based on BLAST statistics (e.g. E-value, coverage, % identity, etc.), users may automate the selection of hits above the Rost’s curve of significant sequence identity (Rost 1999), such as required for structure homology modeling (Krieger *et al.* 2003).

### 3.2 Formatted alignments

Protein sequences and BLAST objects can also be used to generate alignments in different formats. For instance, alignments in PIR format for each hit from searching a query sequence in a database of template structures using BLAST can be used as input for MODELLER (Eswar *et al.* 2008) for homology modeling.

### 3.3 Secondary and super-secondary structures

**Figure 1.**
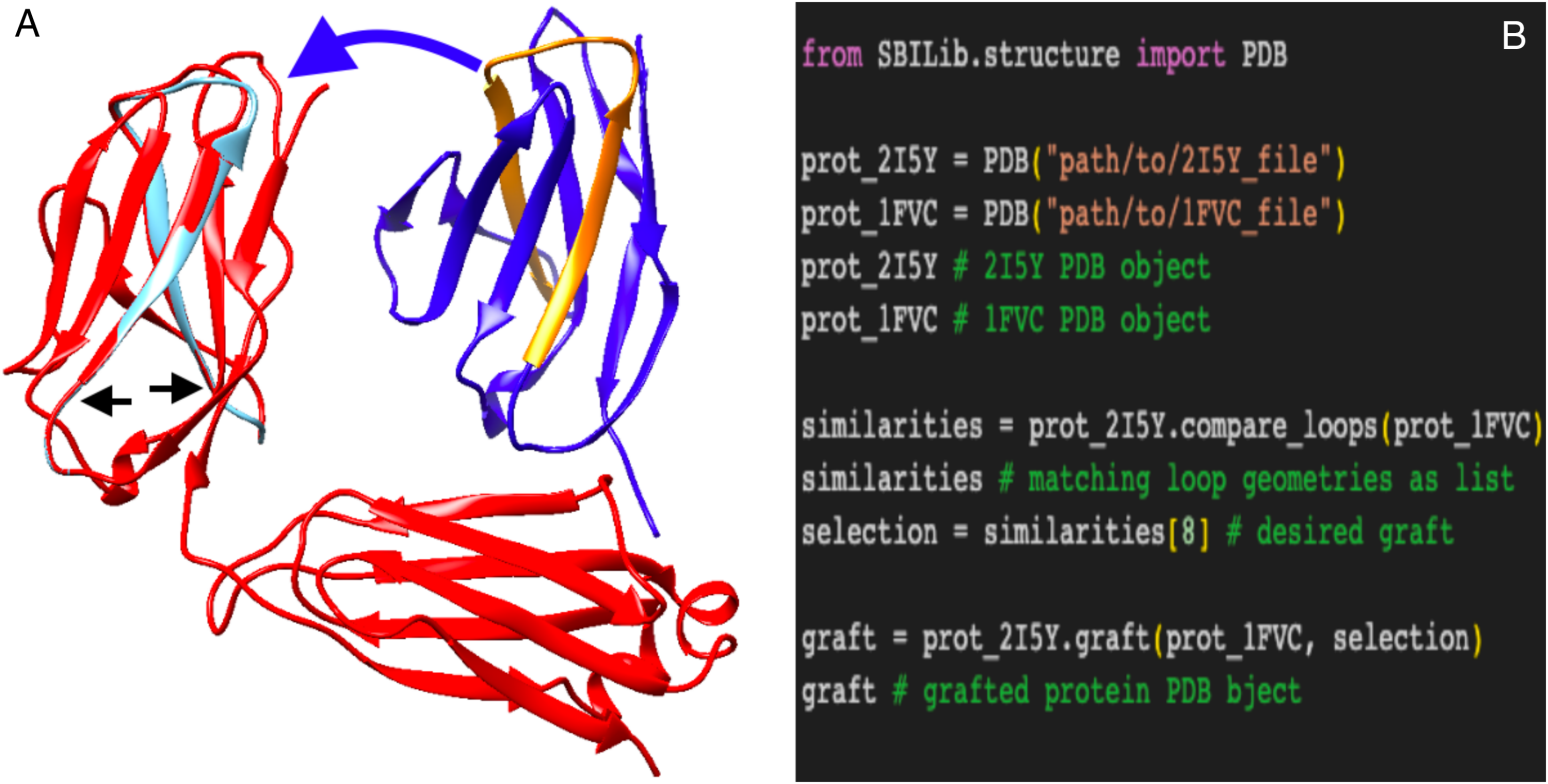
A technical demonstration of SBILib’s grafting function. (A) Anti-HIV-1 antibody 17B (pdb:2I5Y) chain L grafted (red) and ungrafted (light blue) superimposed, and ANTI-P185-HER2 ANTIBODY 4D5 (pdb:1FVC) chain A (dark blue). Due to structural identity only the 2I5Y loop and a few flanking residues are shown in the superimposition (light blue). The light chain loop 1FVC:A:19–38 (orange) was grafted in the place of the light chain loop 2I5Y:L:19–38 (beginning and end marked with black arrows) to produce a grafted protein (red). (B) Workflow followed to produce the grafted protein.

Secondary structure calculation through the integration of DSSP allows SBILib to be used in postmodeling processing of de novo protein structure prediction and the prediction of loops. Protein loops are flexible, disordered regions of a protein connecting two regular secondary structures. Remodeling of the protein backbone for protein design applications often takes place in these regions due to their flexibility. Within SBILib, loop geometry is automatically calculated and can be used to search for similar super-secondary structures using *smotifs* (Fernandez-Fuentes *et al.* 2010). The ability to parse these super-secondary structures is unique to the library. This functionality may be leveraged for downstream applications such as protein loop grafting (Fig. 1).

### 3.4 Protein–protein and protein–DNA interfaces

Residue–residue contacts (between amino acids, nucleotides, or mixed) are handled as lists of objects. Each object has references to positions in the chain object of a PDB object. Distances are calculated using SciPy.

## 4 Examples

The GitHub repository includes several examples describing the use of the library. These include reading protein files in various formats, analyzing basic protein information, handling the results from a BLAST search, viewing the geometry and conformation of smotifs (i.e. features that were used in the classification of loops and their prediction from sequence), as well as investigating protein interactions with other biomolecules (Github: Scenarios.ipynb & README.md).

## 5 Conclusion

SBILib is an open-source Python library for the analysis of protein folds, as well as macromolecular structures and interactions. It can be both implemented programmatically in large bioinformatic projects or used as a stand-alone tool for routine protein structure analyses. The loop handling features of the library can be used in the field of structure-based computational protein design for loop grafting or remodeling purposes. The SBILib package is integral to various current projects and as such is expected to see updates and additions as its functionality within those projects evolve.

## Data Availability

Supplementary information is available at https://github.com/structuralbioinformatics/SBILib. doi: 10.5281/zenodo.8402071.
